# Different effects of Zn nanoparticles and ions on growth and cellular respiration in the earthworm *Eisenia andrei* after long-term exposure

**DOI:** 10.1007/s10646-021-02360-2

**Published:** 2021-02-22

**Authors:** Zuzanna M. Filipiak, Agnieszka J. Bednarska

**Affiliations:** 1 Institute of Environmental Sciences, Jagiellonian University, Gronostajowa 7, 30-387 Kraków, Poland; 2 Institute of Nature Conservation, Polish Academy of Sciences, Mickiewicza 33, 31-120 Kraków, Poland

**Keywords:** Ecotoxicology, Juvenile earthworm, ETS activity, Metal, Soil

## Abstract

In this study, the effects of zinc nanoparticles (ZnO-NPs) and ions (ZnCl_2_) on the mortality, growth, maturation, and cellular respiration of the earthworm *Eisenia andrei* were assessed. Earthworms were individually exposed for 98 days, starting from the juvenile stage, to soils contaminated with either ZnO-NPs or ZnCl_2_ (125, 250, 500 and 1000 mg Zn kg^−1^ dry weight (dw)). Exposure to the highest-concentration ionic treatments (500 and 1000 mg kg^−1^) caused 100% mortality, while for other treatments, mortality did not exceed 15% at the end of exposure. Compared to the control treatment, both 125–1000 mg kg^−1^ ZnO-NPs and 125 or 250 mg kg^−1^ ZnCl_2_ stimulated earthworm growth, which might be due to a hormetic effect. ZnO-NPs and ZnCl_2_ caused different responses at medium Zn concentrations (250 and 500 mg kg^−1^): earthworms exposed to ionic treatment at 250 mg kg^−1^ were characterized by a significantly lower growth constant, lower cellular respiration rate, later inflection point, and higher final body weight than those exposed to ZnO-NPs treatments at the same (250 mg kg^−1^) or twice as high (500 mg kg^−1^) nominal Zn concentrations. However, differences were not observed in all examined parameters between the studied forms when the highest-concentration ZnO-NPs treatment was compared with the lowest-concentration ionic treatment, which was likely due to the same levels of available Zn concentrations in those treatments. Overall, different growth and maturation strategies accompanied by pronounced differences in cellular respiration were adopted by earthworms exposed to low and medium levels of either ZnO-NPs or ZnCl_2_.

## Introduction

Nanoparticle (NP) pollution is a very important issue that has been studied and discussed intensively for the last two decades. Zinc oxide NPs (ZnO-NPs) are among the most commonly used, with wide application in solar panels, paints and coatings, and UV-protection sunscreens (Mirzaei and Darroudi 2017; Sun et al. 2018). Although the predicted environmental concentrations of metallic NPs are very low (Sun et al. 2014) in comparison with those of naturally occurring elements (Kabata-Pendias 1995), prognosis indicates an inevitable increase of NPs in different environmental compartments, including soil ecosystems (Adam and Nowack 2017). Studies on the effects of the NP form of metals on soil invertebrates are of great importance, especially because NPs can be used in fertilizers (Jain et al. 2018).

Concerns about NP toxicity have caused ZnO-NPs to be intensively studied with various standard ecotoxicological tests (Rajput et al. 2018). However, some authors indicate that the present standardized methods proposed by the *Organization for Economic Co-operation and Development* (OECD) or the *International Organization for Standardization* (ISO) might not be suitable for NP ecological risk assessment (Bicho et al. 2015; Waissi-Leinonen et al. 2015). Current test protocols and guidelines developed for soil invertebrates are designed to assess mostly short-term toxic effects of chemicals on endpoints such as the avoidance behavior, survival and reproduction of adult organisms (van Gestel 2012). Nonetheless, examination of adult organisms within a short period of time (relative to their entire lifetime) may lead to underestimation of toxic effects, as the sensitivity of juvenile and adult organisms can differ (Kammenga et al. 1996; van der Ploeg et al. 2011). Long-term studies and more appropriate endpoints have been recognized to provide more ecologically relevant information (Amorim 2016). For instance, a full life cycle test was developed by Bicho et al. (2015) for the soil enchytraeid *Enchytraeus crypticus* to study effects not only on survival and reproduction but also on hatching success and growth. Laskowski (2001), in turn, suggested that whenever accumulation of a chemical throughout the life span of an individual is expected, ecotoxicological experiments should cover at least 2/3 of the total life span.

Different biomarkers can be applied in life cycle studies to obtain a broader understanding of toxic effects (Žaltauskaitė and Sodienė 2014; Das et al. 2018; Lončarić et al. 2020). The electron transport system (ETS) activity in mitochondria can be applied to estimate the intensity of metabolism under particular conditions (Simčič and Brancelj 2006). The ETS activity assessment provides information about the consumption of oxygen that would occur if all enzymes function maximally (note that it does not provide information about oxygen consumption in the whole organism but at the cellular level) (Borgmann 1977; Simčič and Brancelj 2006). It was indicated as a rapid and sensitive method (Fanslow et al. 2001), and since ETS activity is universal to all organisms (Martínez-García et al. 2009), it has been used to determine oxygen consumption for a variety of species (Simčič et al. 2005 and references therein; Bednarska and Stachowicz 2013; Gomes et al. 2015). It is expected that upon exposure to pollutants, the increased energy consumption, i.e., increase in ETS activity, can be associated with enhanced energy demand for defensive and detoxification processes, which in turn cause less energy to be available for growth and reproduction (Calow 1991). On the other hand, at certain point intoxication can lead to diminished ETS activity levels (Gomes et al. 2015) due to impaired mitochondrial respiratory function, which affects the overall functioning of the organism and may lead to death. Overall, this ecologically relevant indicator refers to the energy levels of an organism that constitute an available pool of resources to be allocated into separate and competing “sinks”, e.g., reproduction or detoxification (Calow 1991). Furthermore, our previous study on the energy budget of *E. andrei* adults indicated that ETS activity is a sensitive biomarker of toxic exposure to either ZnO-NPs or ZnCl_2_ (Świątek and Bednarska 2019).

Although the juvenile period is crucial for the population growth rate, only a few authors have focused on the toxic effects of NPs on juvenile earthworms to date. van der Ploeg et al. (2011) examined the C_60_ NP effect on the *L. rubellus* growth rate and effects on the population growth rate after long-term culturing, from the juvenile to adult stage, in contaminated natural soil. The authors showed a decreasing population growth rate with increasing C_60_ concentration (van der Ploeg et al. 2011). Subsequently, Das et al. (2018) proved that compared with controls, juvenile *Eisenia fetida* earthworms cultured for 120 days in natural soil contaminated with Ag NPs exhibited concentration-dependent decreases in survival and body weight with gut histopathological changes and increased enzyme activities at the end of exposure. To the best of our knowledge, the first and so far only attempt to assess the toxic effects of ZnO-NPs on juvenile earthworms was made by Lončarić et al. (2020), who performed a multigenerational test on *Dendrobaena veneta* in which the effects of ZnO in nano or bulk form and the insecticide chlorpyrifos on survival, reproduction, growth rate, and molecular biomarkers were studied. Nevertheless, Lončarić et al. (2020) focused on the toxicity of ZnO in either nano or bulk form mixed with chlorpyrifos, and in our study, we compared the effects of different forms of Zn (ZnO-NPs vs ZnCl_2_) over a wide range of concentrations.

The aim of this study was to determine the impact of Zn as NPs or ions on life cycle parameters, such as survival, growth rate, and maturation, of juvenile *E. andrei* earthworms after long-term exposure to contaminated LUFA 2.2 soil. Additionally, the cellular respiration of earthworms grown in Zn-contaminated soil was determined by measuring ETS activity.

## Materials and methods

### Soil spiking procedure

Standardized loamy sand soil (LUFA-Speyer 2.2, Germany, 2017) was used (see Supplementary Materials for details). ZnO-NPs (25 nm) were used as a powder without any surfactant or coating (PlasmaChem GmbH). The primary particle size and elemental composition were determined previously (Świątek and Bednarska 2019). Soil spiked with zinc chloride salt (ZnCl_2_, Merck Group) was used to represent treatments with ionic Zn. The effects of four concentrations of ZnO-NPs (nominally 125, 250, 500 and 1000 mg Zn kg^−1^ dw soil, designated as ZnO-NPs 125, ZnO-NPs 250, ZnO-NPs 500 and ZnO-NPs 1000, respectively), four concentrations of ZnCl_2_ (nominally 125, 250, 500 and 1000 mg Zn kg^−1^ dw soil, designated as ZnCl_2_ 125, ZnCl_2_ 250, ZnCl_2_ 500 and ZnCl_2_ 1000, respectively), and one control with natural Zn levels in soil were studied. The selected treatments are hereafter referred to as low (125 mg kg^−1^), medium (250 and 500 mg kg^−1^) and high (1000 mg kg^−1^). Moreover, ZnCl_2_ 250 and ZnO-NPs 500 corresponded to concentrations causing a 25% decrease (EC_25_), and ZnCl_2_ 500 and ZnO-NPs 1000 corresponded to concentrations causing a 50% decrease (EC_50_) in earthworm reproduction (Heggelund et al. 2014). For convenience, the studied compounds were introduced into soil using different methods. ZnCl_2_ was applied as an aqueous solution, and ZnO-NPs were dosed as a dry powder, followed by the addition of sufficient water to attain a soil moisture content that was equivalent to 50% of the water-holding capacity (WHC). The spiking procedure is fully described in Świątek et al. (2017). After spiking, each batch of soil was thoroughly mixed using a kitchen robot to achieve a homogenous distribution, and the soil was equilibrated for seven days before the start of the experiment.

### Experimental design

A juvenile toxicity test was designed based on previous studies performed by Spurgeon et al. (2004) and Žaltauskaitė and Sodienė (2014). Twenty replicates (round plastic containers filled with approximately 110 g of wet soil) were prepared per Zn treatment, and thirty replicates were prepared for the control. *E. andrei* earthworms (*N* = 190) were removed from culture medium, washed in tap water, blotted dry, weighed to the nearest 0.0001 g and randomly assigned to treatments at one individual per container. Due to the small size of the worms and their possible mechanical damage while being transferred to experimental containers, containers were checked 8 h after starting the experiment to replace damaged or dead worms with healthy ones. All experimental containers were kept at 20 °C and 75% relative humidity (RH) under a 16:8 (L:D)-h photoperiod for a total of 98 days. Under optimum conditions, the life cycle (from newly laid cocoon to clitellate adult) of *E. andrei* and *E. fetida* is ca. 45–51 days (Domínguez and Edwards 2010). In studies that used artificial soil media, the exposure period ranged between 12 and 14 weeks (van Gestel et al. 1991; Žaltauskaitė and Sodienė 2014); hence, the exposure period in our study lasted for 14 weeks to ensure that the earthworms fully developed. The mortality and growth of earthworms were monitored every fortnight. For surviving earthworms, growth was assessed by weighing the previously washed and blot-dried worms. Sexual development was monitored at days 56, 70, 84, and 98 and recorded following van Gestel et al. (1991): earthworms with a full clitellum were marked as adults, and individuals without this reproductive structure were marked as juveniles. To ensure that the earthworms had sufficient food, horse manure was supplied every second week. At the beginning of the experiment and after 2 weeks, due to the small size of the worms, ca. 0.175 g dw of horse manure was added to each container. The amount of added manure was increased to ca. 0.26 g dw at days 28 and 42 and to 0.35 g dw for all sampling days thereafter. At sampling points, changes in soil moisture were monitored by weighing the containers, and tap water was added if necessary. At the end of the experiment, earthworms were collected, rinsed with tap water, blotted dry on filter paper, and weighed. Then, the animals were kept individually for 24 h in Petri dishes lined with moistened filter paper to void their gut content. Afterwards, the worms were again rinsed with tap water, blotted dry, weighed, frozen in liquid nitrogen and stored at −80 °C until further analysis. Details about *E. andrei* earthworm culture are presented in the Supplementary Materials.

### Cellular respiration rate measurements

The cellular respiration rate was determined by measuring the activity of the ETS as described by Świątek and Bednarska (2019). Earthworms were homogenized on ice using a mechanical Omni tissue homogenizer (TH220-PCR), and measurements were performed in 96-well plates (Sarstedt) using a μQuant spectrophotometer (Bio-Tek Instruments). Earthworms were homogenized in 600 μL of buffer (0.08 M Tris base-HCl (pH 8.5), 15% (w/v) polyvinylpyrrolidone (PVP), 153 µM MgSO_4_, and 0.2% (w/v) Triton X-100). Then, 150 µL of homogenate was centrifuged (1000 × *g*, 10 min, 4 °C) and diluted, if necessary, using ice-cold homogenization buffer. Each sample was analyzed in triplicate. ETS activity was quantified by adding 150 µL of buffered substrate solution (0.13 mM Tris base-HCl (pH 8.5), 0.3% (w/v) Triton X-100, 1.7 mM NADH, 250 µM NADPH) to 50 µL of the resulting supernatant. To start the associated colorimetric reaction, 100 µL of reagent solution (INT) was added, and the absorbance was measured kinetically at 490 nm every 36 s for 3 min at 20 °C. Formazan production was determined by measuring the absorbance of the sample against that of the blank using ɛ = 15900 M^−1^ cm^−1^. The oxygen consumption rate was determined from the ETS activity based on the theoretical stoichiometric relationship that for each 2 µM formazan formed, 1 µM oxygen is consumed by the ETS. The quantity of oxygen consumed was expressed per g body weight (µL O_2_ h^−1^ g^−1^).

### Soil physicochemical analysis

To validate the Zn concentration in the test soil, at the start of the experiment (day 0), soil samples were collected from three randomly selected test containers per treatment. First, soil samples were dried at 105 °C for 24 h, weighed to the nearest 0.0001 g, digested in 10 mL of a 4:1 mixture of HNO_3_:H_2_O_2_ (using the Titan MPSTM system, Perkin Elmer) and ultimately supplemented with 30 mL of demineralized water. Zn concentrations in the solutions were measured using flame atomic absorption spectrometry (AAS, Perkin Elmer AAnalyst 200, detection limit 0.011 mg L^−1^) and expressed in mg kg^−1^ dw. To determine the analytical precision, three blanks and three samples of a certified reference material (Sand 1 CRM048, Sigma-Aldrich; with a certified Zn concentration of 425 ± 9.14 mg kg^−1^) were examined with the soil samples. The measured Zn concentrations in the reference materials were within 16% of the certified concentrations.

Soil samples collected at days 0, 14, 56 and 98 were also analyzed for Zn concentrations in water extracts and pH. The water-extractable Zn concentration was measured after shaking the soil samples with demineralized water (1:4 w/v) (for 2 h at 2000 rpm at room temperature) and filtering through cellulose acetate paper (Eurochem BGD). Afterwards, extracts were acidified with HNO_3_ and analyzed for Zn concentration using flame AAS (Perkin Elmer AAnalyst 200). To determine the analytical precision, blanks and standard solutions with known Zn concentrations were analyzed alongside the samples. The soil pH was measured with 0.01 M CaCl_2_ (1:5 w/v). The soil samples were shaken for 2 h at 2000 rpm at room temperature, and after overnight settling of the floating particles, the pH was measured using a digital pH metre. The soil organic matter content was determined at days 0 and 98 as loss on ignition.

### Data analysis

Distributions of data for all the studied endpoints were assessed for normality with Shapiro–Wilk’s W test, and the homogeneity of variances was analyzed with Levene’s test. If the criteria were not met, the data were either log- or square root-transformed, or a nonparametric test was used. Because all earthworms exposed to ZnCl_2_ 500 and ZnCl_2_ 1000 treatments died before the 56th day, soil was not sampled and analyzed for days 56 and 98. Only individuals that survived until the end of the experiment were considered in the analysis of the endpoints (growth rate parameters, body weight comparisons after exposure, maturity assessment, ETS activity).

The effects of treatment (separately for each sampling day) and time (separately for each treatment) on the Zn concentration in water extracts, soil pH, and the organic matter content were tested using the Kruskal–Wallis test with Bonferroni correction for multiple comparisons. Bonferroni intervals were used to identify differences between treatments or between days at the 95.0% confidence level.

One-way ANOVA was used to verify that the mean initial body mass of earthworms did not differ among treatments before starting the experiment (day 0) and to test the treatment effect on the final body weights (after voiding the gut) of the developed earthworms at the end of the experiment. The initial body weight was not corrected for gut load in the present study, as the contribution of the gut load to the total weight at this developmental stage can be regarded as negligible (Curry and Bolger 1984; Martin 1986). For adult *E. andrei*, the gut load can account for up to ca. 14–16% of the total weight (this study; Arnold and Hodson 2007). There is, however, no consensus on whether the ratio of gut load-to-body weight is fixed under different environmental conditions (Hendriksen 1991; Jager et al. 2003), as this ratio can depend on various factors (Curry and Schmidt 2007). Therefore, although the body weight with the gut load was used to calculate the growth rate during the experiment, the depurated body weight was used to compare final body weights at the end of experiment, as the depurated body weight was also included in our calculations of oxygen consumption (expressed per g body weight).

Survival curves were compared between treatments with the log-rank test, and because significant differences were found (*p* < 0.0001, log-rank test), pairwise comparisons were performed between the control and each Zn treatment, within treatments with the same nominal concentrations (ZnCl_2_ vs ZnO-NPs), and within treatments with the same effective concentrations, i.e., either the EC_25_ (ZnCl_2_ 250 vs ZnO-NPs 500) or the EC_50_ (ZnCl_2_ 500 vs ZnO-NPs 1000).

Individual growth (measured as wet weight) during the experiment was examined by fitting Gompertz growth curves to time series weight values of those earthworms that survived until the end of the experiment. The Gompertz growth curve describes a sigmoidal pattern of growth and has been previously shown to accurately describe earthworm growth (Spurgeon et al. 2004; Žaltauskaitė and Sodienė 2014). The equation adopted from Tjørve and Tjørve (2017) is as follows:

$$W^t = A + (C - A)e^{ - e^{ - B(t - M)}}$$

where *W*^t^ is the weight at time *t*, *A* is the lower asymptotic weight (g), *C* is the upper asymptotic weight (g), *B* is the Gompertz growth constant (day^−1^), and *M* is age at the inflection point (days). The lower asymptotic weight (*A*) was fixed in all calculations based on the worm weight measured at day 0. The maximum growth rate (G_max_, g day^−1^) at the inflection point was calculated as *B*×*C*/e. One-way ANOVA was used to assess the treatment effect on the estimated growth parameters *(B*, *M*, G_max_) and the maturation weight (g), i.e., the weight at which a fully developed clitellum was observed; if significant differences were found, a least significant difference (LSD) procedure was used at the 95.0% confidence level. The maturation time (days), i.e., the first sample point at which a fully developed clitellum was observed, was compared among treatments with the Kruskal–Wallis test, and if significant differences were observed, a Bonferroni procedure was used at the 95.0% confidence level. One-way ANOVA was used to assess the effect of treatments on ETS activity. The data were analyzed statistically using Statgraphic Centurion XVI (StatPoint Technologies, Inc., version 18).

## Results

### Soil total and water-extractable concentrations

The measured total Zn concentrations in the soil were in accordance with nominal values (Table S1), and the total Zn concentration in the control soil was 27.5 ± 1.7 mg kg^−1^ dw (average ± standard deviation, SD; *n* = 3). Significantly lower water-extracted Zn concentrations were observed with the ZnO-NPs 125 treatment than with the ZnCl_2_ 1000 treatment at day 14 (*p* = 0.004). Additionally, significantly lower water-extracted Zn concentrations were observed with ZnO-NPs 125 than with ZnO-NPs 1000 at days 56 and 98 (*p* ≤ 0.008). After the Bonferroni correction for multiple comparisons was applied (*p* ≤ 0.006), the difference in the water-extracted Zn concentrations between days proved to be nonsignificant for each treatment. Nevertheless, in general, a slight decrease in water-extractable concentrations was noted over the 98-day exposure period in all treatments (Table S1).

### Soil pH and organic matter content

The soil pH was predominantly higher in soils contaminated with ZnO-NPs than in those contaminated with ZnCl_2_. The pH was significantly lower with t.0he ZnCl_2_ 500 and ZnCl_2_ 1000 treatments than with the ZnO-NPs 1000 treatment at days 0 and 14 (*p* ≤ 0.002). On day 56 in the control and ZnCl_2_ 125 treatments and on day 98 in the ZnCl_2_ 125 and ZnCl_2_ 250 treatments, the pH was significantly lower than that in the ZnO-NPs 1000 treatment (*p* ≤ 0.005). After applying the Bonferroni correction for multiple comparisons (*p* ≤ 0.006), differences in the pH values between days appeared to be nonsignificant. Nevertheless, in general, a slight increase was observed for ionic treatments, while a decrease followed by an increase in pH levels was observed for NP treatments over the exposure period (Table S2). Regarding soil organic matter content, there were no differences between treatments (*p* = 0.9). Although an increase in organic matter content was observed from day 0 (4.4 ± 0.21%) to day 98 (5.8 ± 0.42%) (average ± SD), after the Bonferroni correction for multiple pairwise comparisons was applied (*p* ≤ 0.006), differences appeared to be nonsignificant for all treatments.

### Mortality

In the ZnCl_2_ 500 and ZnCl_2_ 1000 treatments, mortality reached 100% by days 56 and 28, respectively. Three of 20 earthworms exposed to the ZnO-NPs 500 treatment died (15% mortality), while in the remaining treatment groups, mortality did not exceed 5% during exposure. Survival curves for different exposure conditions (9 treatments) differed significantly (*p* ≤ 0.0001). Pairwise comparisons of survival curves revealed that both ZnCl_2_ 500 and ZnCl_2_ 1000 treatments showed significantly increased mortality in comparison to the control (*p* < 0.0001, log-rank test) and NP treatments with the same nominal concentrations (*p* < 0.0001). No differences in survival curves were found between EC_25_ treatments (ZnCl_2_ 250 vs ZnO-NPs 500) (*p* = 0.08), but the curves for the treatments with concentrations corresponding to the EC_50_ (ZnCl_2_ 500 vs ZnO-NPs 1000) differed significantly (*p* < 0.0001) (Table S3).

### Body weight and growth

The mean body weight of the worms used in the experiment was 0.035 ± 0.0100 g, which did not differ between treatment groups (*p* = 0.2). The final body weights of the earthworms at the end of exposure differed significantly between treatments (*p* = 0.004), with significantly lower body weights observed in the control than in the two ionic treatments (ZnCl_2_ 125 and ZnCl_2_ 250) and significantly higher body weights in the ZnCl_2_ 250 treatment than in all ZnO-NPs treatments (ZnO-NPs 125, ZnO-NPs 250, ZnO-NPs 500, and ZnO-NPs 1000), which did not differ between each other (Table 1).

**Table 1:** Gompertz parameters (*A*—lower asymptotic weight, fixed from data (g), *C*—upper asymptotic weight (g), *B*—Gompertz growth constant (day^-1^), *M*—age at the inflection point (days)), body weight (g), and maturation time (days) of *E. andrei* cultured over the period of 98 days in control soil or soils contaminated with either ZnCl_2_ or ZnO-NPs. Values are presented as the average ± standard deviation

Treatment	Nominal Zn conc (mg kg^−1^)	*A* (g)	*C* (g)	*B* (day^−1^)	*M* (days)	Body weight after 98 days (g)	Maturation time (days)
Control	0	0.037	0.86 ± 0.184	0.024 ± 0.0050^a^	49 ± 13.2^a^	0.54 ± 0.076^a^	87 ± 8.2^a^
ZnCl_2_	125	0.036	0.76 ± 0.177	0.035 ± 0.0118^cd^	36 ± 9.7^cd^	0.60 ± 0.067^bc^	76 ± 17.7^ab^
	250	0.038	0.92 ± 0.185	0.029 ± 0.0057^b^	42 ± 8.5^ab^	0.64 ± 0.064^c^	82 ± 15.9^ab^
ZnO-NPs	125	0.035	0.75 ± 0.106	0.034 ± 0.0059^cd^	34 ± 7.2^d^	0.57 ± 0.063^ab^	66 ± 17.5^b^
	250	0.032	0.81 ± 0.199	0.033 ± 0.0070^cd^	36 ± 8.5^cd^	0.58 ± 0.086^ab^	67 ± 16.6^b^
	500	0.033	0.76 ± 0.124	0.039 ± 0.0091^d^	34 ± 6.4^cd^	0.57 ± 0.099^ab^	81 ± 19.1^ab^
	1000	0.030	0.82 ± 0.226	0.032 ± 0.0115^bc^	40 ± 9.6^bc^	0.57 ± 0.093^ab^	73 ± 16.9^ab^

^a,b,c,d^Different lowercase letters indicate significant differences between treatments

The Gompertz growth constant (*B*; day^−1^) values were significantly higher (*p* < 0.0001) for all Zn treatments than for the control. Growth constants were also significantly higher in the ZnCl_2_ 125, ZnO-NPs 125, ZnO-NPs 250, and ZnO-NPs 500 treatments than in the ZnCl_2_ 250 treatment. Moreover, the *B* values were higher in the ZnO-NPs 500 than in the ZnO-NPs 1000 (Table 1). Thus, significant differences in growth constants were found between EC_25_ treatments (ZnCl_2_ 250 vs ZnO-NPs 500) and between treatments with the same nominal concentration of 250 mg Zn kg^−1^, with higher values for NP treatments than for ionic treatment.

The age of the earthworms at the inflection point (*M*; days), i.e., the point at which the maximum growth rate was observed, was lowest for the ZnO-NP 125 treatment, and this inflection point occurred significantly (*p* < 0.0001) earlier than the inflection point observed for the control, ZnCl_2_ 250 and ZnO-NP 1000 treatments. The highest *M* values were observed for the control, and they were identical only to those of the ZnCl_2_ 250 treatment. Significantly more days to reach maximum growth were needed for worms in the ZnCl_2_ 250 treatment than for worms in the corresponding treatments with the same nominal (ZnO-NPs 250) and effective (ZnO-NPs 500) concentrations (Table 1).

**Fig. 1:**
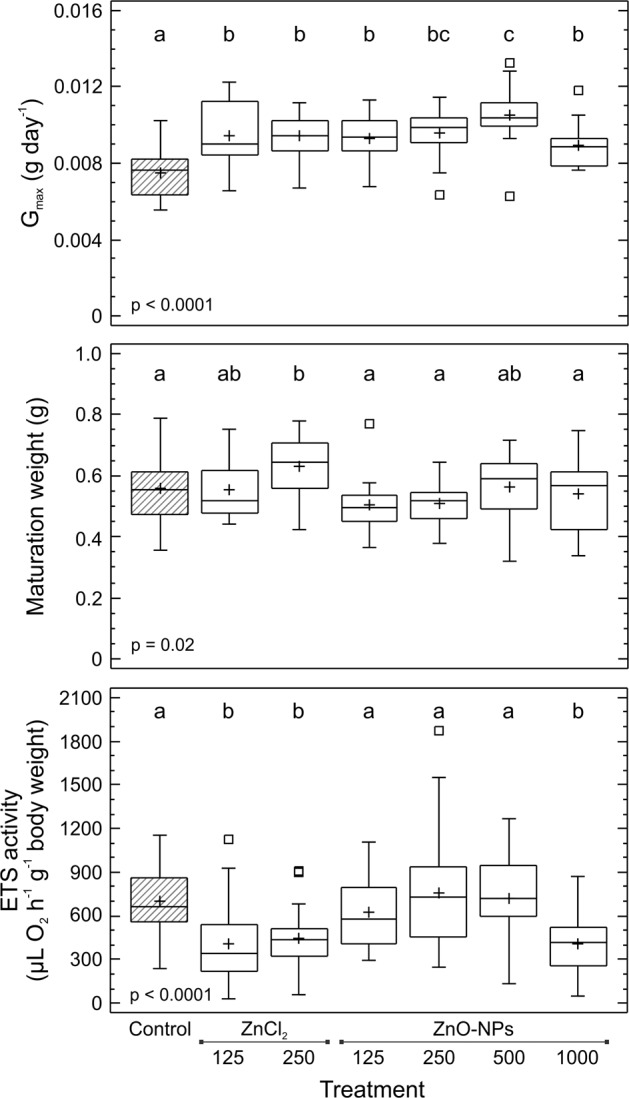
Effects of Zn on the maximum growth rate (G_max_; g day^−1^), maturation weight (g), and electron transport system (ETS) activity (µL O_2_ h^−1^ g^−1^ body weight) in *Eisenia andrei* earthworms cultured on LUFA 2.2 soil contaminated with different concentrations of ZnO nanoparticles (ZnO-NPs) or ions (ZnCl_2_) for 98 days. Boxes—lower and upper quartiles, whiskers extend to the minimum and maximum values, plus sign—mean value, centre line—median, empty squares (outliers)—between >1.5 and 3 times the interquartile range, and dashed box—control. Different letters indicate significant differences (ANOVA; LSD, *p* < 0.05)

The highest maximum growth rate (G_max_, g day^−1^) was observed for worms in the ZnO-NPs 500 treatment, which was significantly higher than the rates for all other treatments except for the ZnO-NPs 250 treatment (*p* < 0.0001). The lowest maximum growth rate was observed for the control, which differed significantly from the rates for all other treatments (Fig. 1). Significant differences in the maximum growth rate were identified between EC_25_ treatments (ZnCl_2_ 250 vs ZnO-NPs 500) but not between treatments with the same nominal concentrations (ZnCl_2_ 250 vs ZnO-NPs 250) (Fig. 1).

### Maturation

By day 56, only 7% of the control earthworms had matured (i.e., the earthworms matured with a developed clitellum), whereas 32 and 20% of worms had matured in the ZnCl_2_ 125 and ZnCl_2_ 250 treatments, respectively. At this point of exposure, the highest percentage of mature earthworms (60%) was found in the ZnO-NPs 125 treatment, followed by 47%, 24%, and 32% in the ZnO-NPs 250, ZnO-NPs 500, and ZnO-NPs 1000 treatments, respectively. At the end of the experiment, in the control, ZnCl_2_ 125, and ZnCl_2_ 250 treatments, 90%, 84%, and 100% of the worms were adults, respectively, while in all ZnO-NPs treatments, 74–80% of the worms were fully developed. Maturation required significantly more time for the control animals than for the worms in the ZnO-NPs 125 and ZnO-NPs 250 treatments (*p* = 0.001), and no other differences between treatments were found (Table 1).

The highest maturation weight was observed with the ZnCl_2_ 250 treatment, while the lowest maturation weight was observed with the ZnO-NPs 125 treatment (*p* = 0.02; Fig. 1). Worms in the ZnCl_2_ 250 treatment had significantly higher maturation weights than those in the control, ZnO-NPs 125, ZnO-NPs 250, and ZnO-NPs 1000 treatments, which did not differ from each other. The maturation weight did not differ between EC_25_ treatments (ZnCl_2_ 250 vs ZnO-NP 500) or between ionic treatments (ZnCl_2_ 125 vs ZnCl_2_ 250).

### ETS activity

ETS activity, i.e., cellular respiration, was similar between ionic (ZnCl_2_ 125 and ZnCl_2_ 250) and ZnO-NPs 1000 treatments but significantly (*p* < 0.0001) lower with these treatments than with all other treatments in which ETS activity was measured (control, ZnO-NPs 125, ZnO-NPs 250, ZnO-NPs 500), which did not differ between each other (Fig. 1).

## Discussion

The present study is the first to compare the responses of earthworm *E. andrei* in terms of mortality, growth, maturation, and cellular respiration to long-term exposure to ZnCl_2_ or ZnO-NPs. The two highest concentrations (500 and 1000 mg kg^−1^) of Zn applied in ionic form caused 100% mortality among juvenile worms after 8 and 4 weeks, respectively, whereas at the same Zn concentrations applied as NPs, mortality did not exceed 15% after 14 weeks of exposure. Our study confirms previous findings showing that ions had higher toxicity than NPs, which was previously reported by several authors for other invertebrates (Notter et al. 2014; Bicho et al. 2017). For instance, Bicho et al. (2017), in their study with *E. crypticus* exposed to CuCl_2_ and CuO nanomaterials in a specially designed full life cycle test, observed 100% mortality of enchytraeids at concentrations of 400 and 800 mg Cu kg^−1^ dw LUFA 2.2 soil when they were exposed to ions and no mortality for those that were exposed to even 1600 and 3200 mg Cu kg^−1^ dw soil in a nanomaterial form.

For ions, 500 mg kg^−1^, i.e., the EC_50_ for reproduction, was lethal to juvenile earthworms, but the same effective concentration in the NP form (1000 mg kg^−1^) caused only 5% mortality at the end of exposure. Moreover, the effects of ZnO-NPs 1000 treatment on growth parameters (*B*, *M*, G_max_), maturation time, and ETS activity were similar to those observed for low-concentration ionic treatments, i.e., ZnCl_2_ 125 and ZnCl_2_ 250. The lack of differences between the ZnO-NPs 1000 and ZnCl_2_ (125 and 250) treatments was likely caused by pH-related processes in the soil matrix that led to similar levels of available Zn in those treatments, thus explaining the equivalent effects in earthworms. In general, the lower the pH is, the higher the concentrations of dissolved metals that are bioavailable to soil-dwelling organisms (Rutkowska et al. 2015). In our study, differences in Zn concentrations were not observed in the water extracts between the ZnCl_2_ treatments and ZnO-NPs 1000 treatment, although the water extraction method might not have accounted for all of the released Zn because water is regarded as a weak extractant (Peijnenburg et al. 2007). However, significantly lower pH values were observed in the ZnCl_2_ 125 treatment at days 56 and 98 and the ZnCl_2_ 250 treatment at day 98 relative to the ZnO-NPs 1000 treatment, which may have promoted equivalent Zn release in the ionic treatments and ZnO-NPs 1000 treatment.

Significantly higher Gompertz growth constants and maximum growth rates were observed for the two ionic treatments (ZnCl_2_ 125 and ZnCl_2_ 250) and for all NP treatments than for the control. Moreover, the time required to develop a clitellum and reach the maximum growth rate was longest in the control earthworms; thus, they were characterized by the latest inflection point among all treatments. Growth stimulation under exposure to metal might indicate that Zn deficiency occurred in the control treatment. Zinc is an essential element that is a component of more than 80 different enzymes operating in a variety of aspects of cellular metabolism, and its deficiency can lead to organism dysfunction (Valko et al. 2005; Rainbow 2007). The concentration of 27.5 mg Zn kg^−1^ dw soil used in our control could be below the optimal concentration for *E. andrei*. A study by Posthuma and Notenboom (1996) indicated that the *E. andrei* earthworm actively assimilates Zn to average levels of ca. 114.7 mg kg^−1^ when exposed to six different field soils with low Zn concentrations ranging between 10.6 and 28.2 mg kg^−1^ dw soil. Moreover, the bioconcentration factors estimated for soils with concentrations above 20 mg Zn kg^−1^ were 3.8–6.6, whereas for soil with 10.6 mg Zn kg^−1^, the factor was 52.4, indicating intensified uptake of Zn upon exposure to low levels of Zn. Several authors have shown that earthworms exposed to soils contaminated with low and moderate levels of different essential elements (Co, Cu) showed increases in body weight, cocoon production, growth, and sexual development relative to earthworms exposed to the control (uncontaminated) soil (Neuhauser et al. 1984; van Gestel et al. 1991; Spurgeon et al. 2003). *Eisenia spp*. earthworms are epigeic species that thrive in organic waste and manure (Domínguez and Edwards 2010), and the Zn concentrations in these materials can reach 245–607 mg Zn kg^−1^ and 79–315 mg Zn kg^−1^, respectively (Ozores-Hampton et al. 1997; Ogiyama et al. 2005; Terzano et al. 2008; Svane and Karring 2019). The texture and physicochemical properties of these media are drastically different from those of Lufa 2.2 loamy sand soil. Although earthworms were fed during the experiment according to the calculation provided by Spurgeon and Hopkin (1996), the Zn levels in the control might have been insufficient. We are unaware of data concerning the effect of zinc deficiency on earthworm growth and development, but generally, insufficient levels of essential elements in the food or medium as well as unbalanced diets have been shown to limit the physiological functions and development of organisms (Lam and Wang 2008; Filipiak 2016, 2019). For instance, a decrease in body size and an increase in mortality were observed for wild bee *Osmia bicornis* developing on Zn-scarce diet (Filipiak and Filipiak 2020).

Cellular respiration (ETS activity) was significantly lower in both ionic (ZnCl_2_ 125 and ZnCl_2_ 250) treatments and the ZnO-NPs 1000 treatment than in the control and the other ZnO-NP treatments. Considering the possible Zn deficiency in the control treatment, whether the cellular respiration rates of the control individuals were physiologically normal is difficult to determine. On the one hand, decreased respiration rates in both the ionic and ZnO-NPs 1000 treatments might be associated with higher toxicity of ionic Zn than ZnO-NPs (Notter et al. 2014) due to impaired mitochondrial function. Nevertheless, such a situation should also be reflected in more severe changes at the whole-body level, e.g., growth arrest, lower body weight or even lethal effects, in comparison with earthworms from other Zn treatments, which was not the case in this study. On the other hand, the respiration rate measured for worms (1) in the control might have been affected by Zn deficiency and increased energy demand for maintenance in this unfavorable condition and that (2) in the ZnO-NPs 125, ZnO-NPs 250, and ZnO-NPs 500 treatments might have been a result of the increased energy demand to deal with oxidative stress (Sonane et al. 2017). Oxidative stress caused by ZnO-NPs in comparison with that caused by ZnCl_2_ was observed after long-term (96 h, considered long-term due to the short life cycle of nematodes) exposure of *Caenorhabditis elegans* to artificial sediment contaminated with either ZnO-NPs or ZnCl_2_ at 2000 mg Zn kg^−1^ (Huang et al. 2019). The authors observed that ZnO-NPs caused significantly higher intracellular ROS generation and gene expression than Zn ions (Huang et al. 2019). This, however, would not explain why earthworms from ZnO-NPs 500 were characterized by the highest Gompertz growth constant (*B*) and one of the youngest ages at the inflection point (*M*), as it is expected that increased expenses for detoxification should lead to diminished levels of energy available for growth (Calow 1991). Alternatively, increased ETS activity in the ZnO-NPs 125, ZnO-NPs 250, and ZnO-NPs 500 treatments might be associated with increased energy demand for, in general, faster maturation (ZnO-NPs 125 and ZnO-NPs 250) and growth (ZnO-NPs 500). Altogether, clear differences were observed in the responses of the worms cultured on soils contaminated with different Zn forms (ZnCl_2_ vs ZnO-NPs), especially at low and medium concentrations.

The enhanced growth (higher values of *B*) and earlier age at the inflection point (*M*) observed for the low ionic (ZnCl_2_ 125) and low and medium NP (ZnO-NPs 125, ZnO-NPs 250, ZnO-NPs 500) treatments compared with that in the control, medium ionic (ZnCl_2_ 250) and high NP (ZnO-NPs 1000) treatments may be associated with a hormetic response. Calabrese and Baldwin (2002) defined hormesis as an adaptive biphasic response distinguished by dose-response characteristics: a low-dose stimulatory response and a high dose inhibitory response. This phenomenon has been observed for metals in ionic form (Calabrese and Baldwin 2003) as well as nanomaterials (Iavicoli et al. 2018; Lead et al. 2018) in a variety of organisms. According to Calabrese and Baldwin (2002), the stimulatory range can be extremely broad (5–100-fold dosage range) and may vary depending on a range of factors; thus, it is not surprising that the low and medium concentrations used in our study caused a stimulating effect. Faster growth and larger body sizes have been recorded at low levels of ZnO-NPs (0.125 mg L^−1^) compared with those in the control and high ZnO-NP levels in *Xenopus laevis* tadpoles (Nations et al. 2011). Nations et al. (2011) argued that given the essential nature of Zn, the term hormesis may not be applicable for the observed dose response phenomenon. However, Jager et al. (2013) did not exclude a situation in which hormesis occurs under exposure to essential metals, and includes such in the medication category. Therefore, the medication category of hormesis does not preclude Zn deficiency. Apart from medication, the authors also distinguish two other categories: acquisition and allocation (Jager et al. 2013). Most importantly, the authors emphasized that the conservation laws for mass and energy must be obeyed in organisms; thus, hormesis is related to trade-offs between traits, i.e., an increase in the performance of one trait leads to a decrease in performance in another trait. The trade-offs indicated by Jager et al. (2013) were observed to some extent in our study because different patterns of growth, energy consumption and maturation were observed for NPs and ions, especially for treatments with the same medium nominal concentration (ZnCl_2_ 250 vs ZnO-NPs 250) or EC_25_ treatments (ZnCl_2_ 250 vs ZnO-NPs 500). In general, the faster growth and maturation in the low- and medium-ZnO-NPs treatments relative to that in the ZnCl_2_ 500 treatment were probably associated with increased energy turnover, which was indicated by the increased oxygen consumption measured as ETS activity. Earthworms from the ZnCl_2_ 250 treatment achieved the maximum growth rate gradually, and their inflection point (*M*) and growth constant (*B*) were the lowest (from all Zn treatments); however, these worms were characterized by the highest final body weight. Moreover, even though the worms in the ZnO-NPs 250 and ZnO-NPs 500 treatments had the highest maximum growth rate, only three-quarters of them reached sexual maturity at the end of exposure. In contrast, all worms from the ZnCl_2_ 250 treatment, which had relatively low growth parameters, fully developed a clitellum during the experiment. Clearly, hormesis-like effects might only be a characteristic of the early stage of life and may not incur visible trade-offs at later stages because short-term stimulatory effects may not necessarily affect populations in the long term (Tyne et al. 2015). On the other hand, we cannot exclude that the patterns observed in the present study for NPs and ions at the juvenile and young adult stages may be reflected in subsequent life stages, for example, as differentiated reproductive outputs (Spurgeon et al. 2003). Therefore, in the next step, the effects observed here should be evaluated under other experimental conditions to answer the questions that arose in this study.

## Conclusions

Overall, the obtained results indicate different responses of *E. andrei* earthworms, from newly hatched to fully developed earthworms, exposed to ZnO-NPs and ions for long periods of time. Higher toxicity was observed for ZnCl_2_ than for NPs at the same nominal concentrations: exposure to ZnCl_2_ 500 and ZnCl_2_ 1000 resulted in 100% mortality. Distinct differences in growth and maturation were observed between the control and Zn-treated earthworms, and the fact that the control performed worse can be explained by the hormetic effect. The lack of differences in growth parameters (*B*, *M*) and cellular respiration between ionic treatments (ZnCl_2_ 125 and ZnCl_2_ 250) and the highest NP treatment (ZnO-NPs 1000) were most likely associated with similar levels of available Zn concentrations in those treatments. Different response patterns observed for earthworms exposed to either ZnO-NPs or Zn ions were reflected in the different growth and maturation strategies accompanied by pronounced differences in metabolism, i.e., cellular respiration, especially visible between treatments with the same nominal concentrations (ZnCl_2_ 250 vs ZnO-NPs 250) and EC_25_ treatments (ZnCl_2_ 250 vs ZnO-NPs 500).

## Supplementary information

Supp_material_Growth_minor-rev-final

## Data Availability

Data, associated metadata, and calculation tools are available from the corresponding author.
