# Supplementary material for: Different effects of Zn nanoparticles and ions on growth and cellular respiration in the earthworm Eisenia andrei after long-term exposure
Source: Ecotoxicology. 2021 Feb 22;30(3):459–69. doi: 10.1007/s10646-021-02360-2 (PMC7987695; doi:10.1007/s10646-021-02360-2)
Supplement: Supplementary file 1 — Supp_material_Growth_minor-rev-final [file 10646_2021_2360_MOESM1_ESM.docx]

**SUPPLEMENTARY MATERIAL FOR:**

**Different effects of Zn nanoparticles and ions on growth and cellular respiration in the earthworm *Eisenia andrei* after long-term exposure**

**Ecotoxicology**

Zuzanna M. Filipiak^a*^, Agnieszka J. Bednarska^b^

^a^Institute of Environmental Sciences, Jagiellonian University, Gronostajowa 7, 30-387 Kraków, Poland, ORCID: 0000-0001-5843-5195

^b^Institute of Nature Conservation, Polish Academy of Sciences, Mickiewicza 33, 31-120 Kraków, Poland, ORCID: 0000-0002-1030-3414

***CORRESPONDING AUTHOR**

E-mail address: zuzanna.filipiak@alumni.uj.edu.pl

**MATERIALS AND METHODS**

***Soil characteristics***

The reported physicochemical properties of LUFA 2.2 soil were pH_CaCl2_ 5.6 ± 0.4, total organic carbon content 1.73 ± 0.27%, cation exchange capacity 9.8 ± 0.5 meq 100 g^-1^, and maximum water-holding capacity (WHC) 45.8 ± 1.9% (w/w).

***Test species***

Earthworms of the species *E. andrei* were obtained from a laboratory culture kept in the Institute of Environmental Sciences, Jagiellonian University, in Kraków, Poland. The earthworms were fed horse manure free of any pharmaceuticals and cultured at 20°C in darkness. The experiment used 2- to 3-week-old juvenile individuals with an average (± standard deviation, SD) weight of 0.035 ± 0.0100 g.

**Table S1.** Total and water-extractable zinc concentrations (average ± standard deviation) in LUFA 2.2 soil at different nominal Zn concentrations applied as ZnCl_2_ or ZnO-NPs; water extracts collected at days (t): 0, 14, 56 and 98.

| Treatment | Total Zn concentration in soil (mg kg^-1^) | | Zn concentration in water extracts (mg L^-1^) | | | |
| --- | --- | --- | --- | --- | --- | --- |
|  | Nominal | Actual | t = 0  (N = 3) | t = 14  (N = 3) | t = 56  (N = 3) | t = 98  (N = 3) |
| **Control** | **0** | 27.5 ± 1.7 | <LOD | <LOD | <LOD | <LOD |
| **ZnCl_2_** | **125** | 147.6 ± 1.2 | 0.9 ± 0.09 _A_ | 0.9 ± 0.16 _AB_ | 0.6 ± 0.08 _AB_ | 0.6 ± 0.10 _AB_ |
|  | **250** | 263.7 ± 4.9 | 3.1 ± 0.30 _A_ | 2.3 ± 0.30 _AB_ | 1.3 ± 0.19 _AB_ | 1.5 ± 0.30 _AB_ |
|  | **500** | 524.6 ± 94.9 | 5.4 ± 0.26 _A_ | 4.9 ± 0.13 _AB_ | NA | NA |
|  | **1000** | 1083.6 ± 61.5 | 8.1 ± 0.33 _A_ | 8.1 ± 0.44 _B_ | NA | NA |
| **ZnO-NPs** | **125** | 129.5 ± 1.4 | 0.7 ± 0.28 _A_ | 0.5 ± 0.15 _A_ | 0.4 ± 0.01 _A_ | 0.5 ± 0.14 _A_ |
|  | **250** | 278.0 ± 20.5 | 1.2 ±0.08 _A_ | 1.0 ± 0.09 _AB_ | 0.8 ± 0.05 _AB_ | 0.8 ± 0.19 _AB_ |
|  | **500** | 529.4 ± 27.3 | 2.8 ± 0.15 _A_ | 2.3 ± 0.66 _AB_ | 1.3 ± 0.29 _AB_ | 1.6 ± 0.30 _AB_ |
|  | **1000** | 1041.8 ± 29.2 | 4.6 ± 0.66 _A_ | 3.9 ± 1.08 _AB_ | 3.0 ± 1.59 _B_ | 3.0 ± 0.6 _B_ |

A, B – Different uppercase letters indicate significant differences between treatments for each day; Kruskal-Wallis test with a 95.0% Bonferroni confidence interval (p ≤ 0.0125 after Bonferroni correction for multiple comparisons); <LOD – below limit of detection; NA – data not available due to high mortality in those treatments.

**Table S2.** pH_CaCl2_ (average ± standard deviation) of LUFA 2.2 soil in control and different treatments with ZnCl_2_ or ZnO nanoparticles (ZnO-NPs), measured at days (t) 0, 14, 56 and 98.

| Treatment | Nominal Zn concentration (mg kg^-1^) | pH in soil |  |  |  |
| --- | --- | --- | --- | --- | --- |
|  |  | t = 0  (N = 3) | t = 14  (N = 3) | t = 56  (N = 3) | t = 98  (N = 3) |
| **Control** | **0** | 5.9 ± 0.02 _AB_ | 6.0 ± 0.03 _AB_ | 5.7 ± 0.04 _A_ | 6.2 ± 0.06 _AB_ |
| **ZnCl_2_** | **125** | 5.7 ± 0.01 _AB_ | 5.9 ± 0.04 _AB_ | 5.6 ± 0.13 _A_ | 6.1 ± 0.06 _A_ |
|  | **250** | 5.5 ± 0.02 _AB_ | 5.8 ± 0.04 _AB_ | 5.9 ± 0.02 _AB_ | 6.1 ± 0.11 _A_ |
|  | **500** | 5.2 ± 0.03 _A_ | 5.6 ± 0.07 _A_ | NA | NA |
|  | **1000** | 5.1 ± 0.05 _A_ | 5.5 ± 0.09 _A_ | NA | NA |
| **ZnO-NPs** | **125** | 6.4 ± 0.01 _AB_ | 6.2 ± 0.07 _AB_ | 6.1 ± 0.02 _AB_ | 6.4 ± 0.03 _AB_ |
|  | **250** | 6.4 ± 0.03 _AB_ | 6.2 ± 0.05 _AB_ | 6.2 ± 0.04 _AB_ | 6.4 ± 0.02 _AB_ |
|  | **500** | 6.4 ± 0.02 _AB_ | 6.2 ± 0.06 _AB_ | 6.3 ± 0.02 _AB_ | 6.5 ± 0.02 _AB_ |
|  | **1000** | 6.7 ± 0.01 _B_ | 6.4 ± 0.04 _B_ | 6.6 ± 0.03 _B_ | 6.6 ± 0.01 _B_ |

A, B – Different uppercase letters indicate significant differences between treatments on each day; Kruskal-Wallis test with 95.0% Bonferroni confidence level (p ≤ 0.0125 after Bonferroni correction for multiple comparisons); NA – data not available due to high mortality in those treatments.

**Table S3.** Pairwise comparison of survival curves for *E. andrei* earthworms after long-term (98 days) exposure to control soil and soils contaminated with different concentrations of Zn applied as ZnCl_2_ or ZnO-NPs. Log-rank test, *chi-square* value and *p-value*.

| Comparison | Treatments | *chi-square* | *p-value* |
| --- | --- | --- | --- |
| **control vs ZnCl_2_** | control vs ZnCl_2_ 125 | 0.0001 | 1.0 |
|  | control vs ZnCl_2_ 250 | 0.7 | 0.4 |
|  | control vs ZnCl_2_ 500 | 54.1 | < 0.00001 |
|  | control vs ZnCl_2_ 1000 | 46.0 | < 0.00001 |
| **control vs  ZnO-NPs** | control vs ZnO-NPs 125 | 0.7 | 0.4 |
|  | control vs ZnO-NPs 250 | 0.1 | 0.8 |
|  | control vs ZnO-NPs 500 | 2.1 | 0.2 |
|  | control vs ZnO-NPs 1000 | 0.1 | 0.8 |
| **nominal vs nominal** | ZnCl_2_ 125 vs ZnO-NsP 125 | 0.7 | 0.4 |
|  | ZnCl_2_ 250 vs ZnO-NPs 250 | 1.0 | 0.3 |
|  | ZnCl_2_ 500 vs ZnO-NPs 500 | 43.3 | < 0.00001 |
|  | ZnCl_2_ 1000 vs ZnO-NPs 1000 | 38.3 | < 0.00001 |
| **EC_25_ vs EC_25_** | ZnCl_2_ 250 vs ZnO-NPs 500 | 3.2 | 0.08 |
| **EC_50_ vs EC_50_** | ZnCl_2_ 500 vs ZnO-NPs 1000 | 43.3 | < 0.00001 |
